# Improved anti-biofouling resistances using novel nanocelluloses/cellulose acetate extracted from rice straw based membranes for water desalination

**DOI:** 10.1038/s41598-022-08324-8

**Published:** 2022-03-14

**Authors:** Ashraf Morsy, Amira S. Mahmoud, Aya Soliman, Hesham Ibrahim, Eman Fadl

**Affiliations:** 1 Department of Materials Science, Institute of Graduate Studies and Research, Alexandria University, Alexandria, Egypt; 2 Petrochemicals Department, Faculty of Engineering, Pharos University, Alexandria, Egypt; 3 Department of Environmental Studies, Institute of Graduate Studies and Research, Alexandria University, Alexandria, Egypt

**Keywords:** Environmental sciences, Materials science, Nanoscience and technology

## Abstract

Cellulose and Nanocellulose acetate (NCA) have attractive novel properties like excellent mechanical properties, rich hydroxyl groups for modification, and natural properties with environmental friendliness. Cellulose was extracted from rice straw wastes as an extra value, then it had been further transformed into NCA using the acidic hydrolysis technique. The structural, crystalline, morphological, were characterized by Fourier transform infrared spectroscopy (FTIR), Proton nuclear magnetic resonance (^1^HNMR), X-ray diffraction (XRD), Scanning microscopy, respectively. The particle size of the Nanocellulose extracted from rice straw was about 22 nm with a spherical shape. Development membranes were prepared with different concentrations of NCA to improve the performance and the anti-biofouling properties of cellulose acetate reverse osmosis (RO) membranes using a phase inversion technique. The structural of membranes were characterized by FTIR, water contact angle measurements, while the anti-biofouling properties were studied by static protein adsorption. The results indicated the development membrane features a lower contact angle accomplished with exhibits pore-forming ability and enhanced hydrophilicity of prepared membrane, furthermore the development cellulose acetate reverse osmosis (CA-RO) membranes with 40:60% RNCA:CA produced a salt rejection of 97.4% and a water flux of 2.2 L/m^2^ h. the development membrane have resists effectively protein adsorption and microbial growth showed from the results of Static protein adsorption.

## Introduction

The persistent use of polymers constitutes a significant source of environmental pollution, harming wildlife when they are dispersed in nature^1^. Increasing concern exists today about the preservation of ecological systems. However, most of today’s synthetic polymers are produced from petrochemicals and rarely are biodegradable^2^. In Egypt, the rice cultivation produces large amounts of rice straw as residues. Assuming that about 20% of rice straw is used for other applications, about 2.8 Mt stay in the fields to be burnt within a time of 30 days to quickly get rid of leftover debris which resulting emissions produce significant pollution to the air, called the “Black Cloud”^3^. As a result, only an estimated 20% of the total annual rice residues output is utilized both on and off-farm^4^. Cellulose is the most common natural polymer isolated from rice straw which contains about 45–70% complex carbohydrates (cellulose and hemicellulose)^5,6^. Owing to its own unique properties, compared to its conventional usage, Nanocellulose (NC), with a crystalline structure, can be used in various industrial applications as a novel sustainable future material^7,8^. Aqueous suspensions of cellulose nanoparticles can be prepared by the mechanical treatment of cellulosic biomass. This cellulosic material consists of highly crystalline residue that may be converted into a stable suspension by mechanical shearing action, but it did not convert it to cellulose acetate as one application^9^. The most common membrane material used for water application is considered cellulose acetate (CA) due to its natural characteristics, low cost, extraordinary potential flux^10^. Cellulose acetate membranes are restricted application because of the buildup of biological matter at the membrane surface; considered as biofouling will be an accumulation of Bioorganic matter onto the surface of the membranes an irreversible deposition. The attachment of microorganisms to the membrane surface, following the growth of colonies on the surface considered a kind of fouling^11^. Previous studies indicated that the physicochemical properties of the membrane surface, are major factors influencing fouling like roughness, charge and hydrophilicity^12^. The effective route to develop anti-fouling membranes possible by surface modification and structuring of polymer surfaces could even be an easy tool of existing membranes^13^. This method some researchers adopted to modify the surface properties of water filtration membrane^14^. Therefore, it’s very important, but also a challenge to improve the anti-bacterial activity of CA-RO membranes for desalination. The basic route in CA anti-bacterial modification is the introduction of anti-bacterial materials or groups into membrane matrix through various techniques, for instance, physical blending technology, introducing inorganic particles, organic anti-bacterial polymers and natural polymer agents into CA membranes^15^. The enhanced surface hydrophilicity of the membranes results in the improvement of their antifouling performance using modification techniques for the membrane surface such as free radical, photochemical, radiation, coating, and plasma-induced grafting. The antibacterial quaternary ammonium groups have been covalently grafted onto the CA-RO membrane surface by of 2-acrylamido-2-methylpropanesulfonic acid in our previous study^16^. The enhancements of permeability and salt rejection in membrane depend on nanoparticles used that have smaller dimensions are an ongoing research prospect for improving membrane performances^17^. The carbonaceous nanomaterials, carbon dots (CDs) as an emerging has drawn tremendous attention due to their low toxicity, ease of preparation, and functionalization^18^. The face parcels and size of CDs nanoparticles can be fluently tuned by changing the raw material or response parameters^19^. The number of functional groups, similar as carboxyl, carbonyl, and hydroxyl, amino, and other oxygenous groups makes it largely answerable and biocompatible to enhance the hydrophilicity of membrane surface^20^. Cellulose acetate membranes with Linde type A (LTA) Zeolite Nanoparticles (ZNPs) were investigated as mixed matrix membranes (MMMs) increased the contact angle of the membranes slightly with maintaining the hydrophilic nature of the MMMs^21^. The main idea of this work is to extract Cellulose from rice straw and transform it to Nanocellulose acetate (NCA) and improve the performance of CA-RO membrane and anti-fouling properties by adding (NCA) to a membrane using the phase-inversion technique. The development membranes will be characterized by Fourier transform infrared (FTIR), contact angle, scanning electron microscopy (SEM) techniques. The pristine and development membranes will be assessed for the performance of water desalination using water flux and salt rejection.

## Materials and methods

### Materials

Solid agricultural wastes (rice straw) were obtained from the local farms (Our study complies with relevant institutional, national, and international guidelines and legislation), Acetone, Sodium hydroxide pellets, and Glacial acetic acid were purchased from CARLO ERBA, Acetic anhydride, and Sulfuric acid was obtained from Fisher Scientific. Chloroform and Formamide were received from Aldrich. Methanol was used as obtained from Labsolve (Lisbon, Portugal) and the polyester sheet Nonwoven Fabric with thickness 120 µm (85 g/m^2^)(Novatexx 2484) was purchased from Freudenberg Filtration Technologies Company,Germany.

### Extraction and preparation of nanocellulose from rice straw

The Cellulose was extracted from rice Straw and converted to Nanocellulose according to the method with some modifications^22^. The rice straw was grinded by a Wiley mill and hydrolyzed with applied. The rice straw was mechanically stirred with High-speed Refrigerated Centrifuge (CR22GIII) at a ratio of 1:20 (w/v) of aqueous concentrated sulfuric acid (40% w/w) with a Teflon bar dispersing element, at 45 °C for 60 min. The Nanocellulose suspension was diluted tenfold with deionized water to quench the reaction and it was centrifuged at 6000 rpm for 10 min to concentrate the Nanocellulose and to remove excess acid. The precipitate was resuspended in distilled water and dialyzed with distilled water until a pH (6–7) was reached by using pH/Conductivity Meter (TDS) (430 portable, Jenway, England). The process of centrifugation through dialysis was repeated three times, then NaOH (15%) was added to the solution at 45 °C for 60 min to remove lignocellulose. The liquids were washed once with three volumes of water and dried in the oven at 60 °C.

### Acetylation of nanocellulose

**Figure 1:**
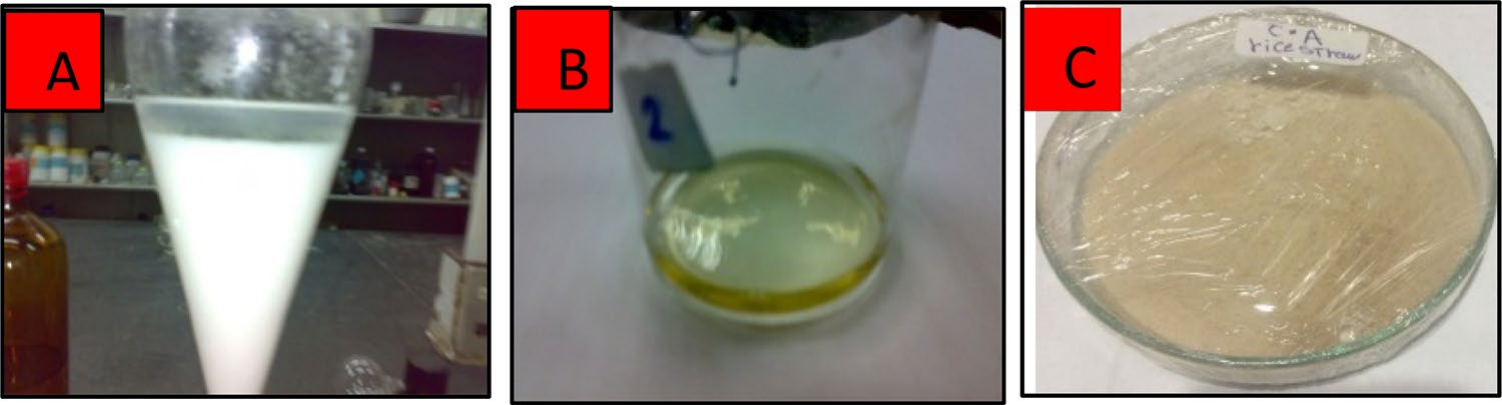
Steps of preparation of NCApowder. ((**A**) Activation of Nanocellulose with acetic acid, (**B**) Complete acetylation, (**C**) Precipitation and drying of NCA).

acid in a 250 mL beaker. The mixture was placed in a water bath with temperature maintained at 50–55 °C for 30 min with continuous stirring. The acetylating mixture, consisting of 10 mL of acetic anhydride and 0.4 mL of concentrated sulfuric acid, was added to the above reaction mixture. The reaction mixture was then kept in a water bath for one hour at 50–55 °C with continuous stirring until a clear solution was obtained. Added 24 mL of acetic acid and 7 mL water were added to the mixture with stirring to avoid precipitation. The mixture was allowed to stand for 1 h at 55 °C and poured into a large volume of water. After the Nanocellulose acetate (NCA) precipitate was formed, filtered through a Buckner funnel, then washed to neutrality, and dried in air^23^. Figure 1 shows the preparation steps of NCA powder.

### Preparation of CA-RO membranes by phase inversion technique

Cellulose acetate reverse osmosis (CA-RO) membrane was prepared using a mixture of methanol (8 g) was used as a non-solvent, acetic acid (5 g), and acetone (10 g) as solvents and dioxane (27 g) additionally to different ratios (35, 40, 50, 100) wt% of NCA with CA the whole of the mixture (8 g) as presented in Table 1. This mixture of the solution was left under stirring for 24 h at room temperature until the CA was completely dissolved. The polymer solution was put into an ultrasonic bath for 45 min to remove the air bubbles entrapped within the solution. The knife of an automatic applicator using to get the CA-RO membranes through the spreading of the solution onto a polyester sheet nonwoven on a glass plate at room temperature. The automatic film applicator (Zehntner 2300-Swiss) using at a constant speed of casting (10 mm/s)and the thickness of the membrane was selected (250 μm). After casting the polymer solution with an evaporation time of the solvent (dioxane, acetone) of 60 s, the CA membrane (cast onto the glass plate) was immersed for 15 min in a deionized water ice bath. The CA membrane formed was placed in a water bath at about 4 °C for 2 h and washed with distilled water to completely remove any residual solvents. The post-treated then of CA-RO membranes for 10 min at about 80–85 °C and then immersed in deionized water for 24 h and air-dried for 24 h before characterization^24^.

**Table 1:** The percentage mixture of RNCA and CA for studied membranes.

CA membrane (%)	RNCA membrane (%)	RNCA: CA membrane (%)	RNCA: CA membrane (%)	RNCA: CA membrane (%)
100	100	50: 50	40:60	35:65

### Characterization techniques

The particle size of the Nanocellulose powder was studied using scanning electron microscopy (SEM) (Joel JSM 5300, Japan EM). Samples dried powder was mounted on a brass plate and the sample film was then sputter-coated with a thin film of gold. Samples were prepared as follows. The dried membranes were cut under liquid nitrogen treatment that was used to give a generally consistent and clean break of the membraneand mounted on a brass plate. The membranes were then sputter-coated with a thin film of gold. The degree of substitution (DS) of the NCA was determined by ^1^HNMR spectroscopy (JEOL GNM ECA 500 spectrometer) at room temperature after adding a drop of trifluoroacetic acid whose function is to shift the active hydrogen to a low field area, and the DS of the CA was calculated by relating the peak areas of the acetyl protons to the total areas of protons. The spectrum of the cellulose ester was measured in deuterated solvent such as dimethyl sulfoxide (DMSO). FTIR spectroscopy (Perkin Elmer BXII) is a powerful analytical technique for qualitative and semi-quantitative analysis. The intensity of the incident radiation FTIR spectrum is recorded in the wavenumber range of 400–4000 cm^−1^for the prepared CA and CA-RO membranes. X-ray diffraction (XRD) (X-ray 7000 Schimadzu-Japan) scans were carried out at room temperature where the Bragg angle (2θ) ranges from 5 to 80 degrees to determine the degree of crystallinity in the prepared disk. The X-ray source was a Cu target setting of 30 kV and 30 MA, with a scan speed of 4 deg/min^25^.The hydrophilicity measurements of the surface of the membrane were measured using Rame-Hart, Instrument Company, France. A drop of distilled water of about 2 μL was placed on the membrane surface (3 × 2 cm) using a microsyringe (Hamilton Company, Reno, NV). The contact angle was measured within 20 s after the water drop was placed. To improve the statistics of the contact angle measurement, each reported data point is the average of five measurements at different positions at the same condition. The performance tests (salt rejection and water flux) for the membrane sample (area 42 cm^2^) were done using a cross-flow RO unit (CF042, Sterling, USA). A flow meter F-550 (USA) was connected to obtain a constant flow of 1 L/min. The membranes were flushed in the RO unit system with deionized water for 30 min until a steady permeate flux was achieved, then the saline salt solutions of NaCl of 10,000 ppm was introducedand. The pH and the temperature of the feed solution were kept constant at 7 and 25 °C, respectively The determination of the total dissolved salt of the permeate water was measured with a pH and conductivity meter (430 portable, Jenway, England). The water flux (F) and salt rejection (R) values were obtained using Eqs. (1), (2):

1 $${\text{F}} = \frac{{\text{V}}}{{{\text{A}} \times {\text{t}}}}$$

where V is the total volume of water passing through the membrane (L), A is the membrane area (m^2^), and t is the time (h).

2 $${\text{R}} = ({\text{C}}_{{\text{o}}} {-}{\text{C}}_{{{\text{memb}}}} ) \times \frac{100}{{{\text{Co}}}}$$

where Co is salt concentration in the feed water side and Cmemb. is the salt concentration in the permeate side of the membrane. For anti-biofouling test, Egg albumin powder (1 gm) was dissolved in a buffer solution (pH = 7.1). To test the protein adsorption, membranes were cut into pieces 2.5 × 2.5 cm in size, and 2 pieces of the samples were put into a glass vial containing 10 mL of the egg albumin as a protein feed solution. The vials were vibrated in a shaking bath at a constant temperature of 25 °C for 24 h to reach the protein adsorption equilibrium^10^. The amount of adsorbed protein was determined by measuring the concentration of the protein solution before and after adsorption. The concentration of protein was measured using UV spectrophotometer (UV-1601, Shimadzu, Japan). The relative protein adsorbed (RP%) was used to identify the extent of adsorptive fouling obtained from Eq. (3) ^26^:

3 $${\text{RP}}\% \, = \frac{{\left( {{\text{C}}_{{\text{o}}} { }{-}{\text{ C}}_{{\text{a}}} { }} \right)}}{{{\text{C}}_{{\text{o}}} }} \times 100$$

where Co and Ca are protein concentrations before and after membrane soaking, respectively. The integrated ratio of the seven cellulose protons resonating in the range of 3.5–5.1 ppm relative to the integrated absorbance of the three methyl protons of the acetyl groups resonating in the range of 1.9–2.2 ppm determines the degree of substitution (DS) of CA according to the Eq. (4):

4 $${\text{DS}} = \frac{{7 \times I_{acetyl} }}{3 \times LH,AGU}s$$

where I_acetyl_ is the integration of methyl protons of the acetyl moieties, I_AGU_ is the integration of all protons of the anhydroglucose unit, numbers 7 and 3 indicate the cellulose seven protons and the three methyl protons of the acetyl moieties respectively. The distribution of the acetyl moiety among the two OH groups of the AGU of cellulose acetate was calculated from the spectra^27^.

### Human and animal participants

This article does not contain any studies involving animals’ studies or human participants performed by any of the authors.

## Results and discussion

### Morphological properties of prepared the Nanocellulose powder

**Figure 2:**
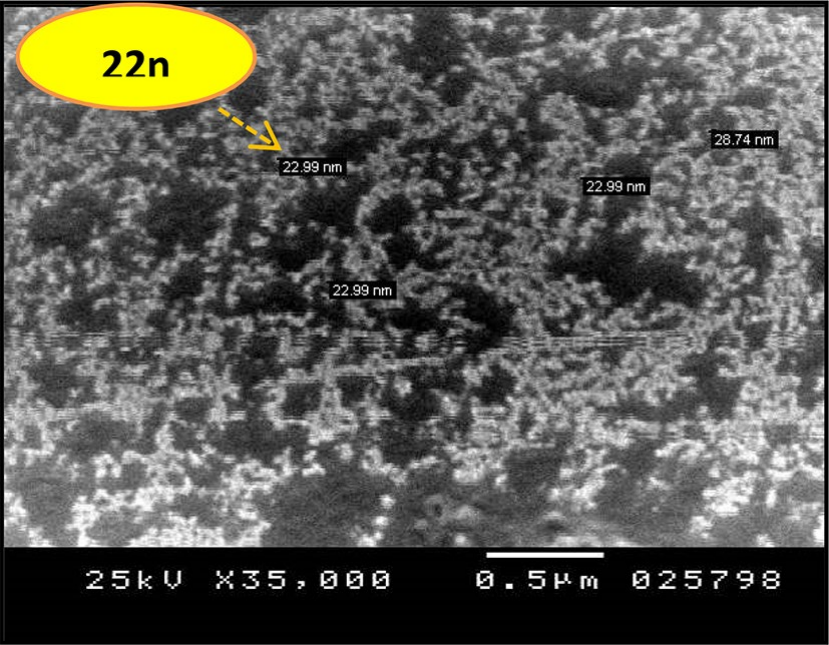
SEM image of Nanocellulose extracted from rice straw.

The morphology and particle size of prepared the Nanocellulose powder from Rice straw, was investigated by the SEM image and was shown in Fig. 2. The particle size of Nanocellulose extracted from the rice straw was about 22 nm with a spherical shape which is in agreement with Shi and Liu^28^.

### Mechanism of acetylation of the nanocellulose

**Figure 3:**
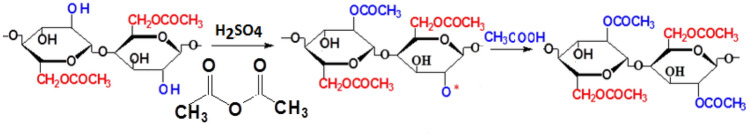
A Reaction mechanism to obtain a cellulose diacetate (CDA) from cellulose.

In the first step of the acetylation process, the acetic anhydride extracts a proton (a hydrogen ion) from the concentrated sulphuric acid, which is more favored than the perchloric acid (HClO_4_) for the pristine process because sulphuric acid is economy and it does not present a potential hazard associated with a buildup of perchlorates (ClO_4_^−^) in the acid recovery system. The proton is then attached to one of the lone pairs on the oxygen of cellulose, which is double-bonded to the carbon. A proton with a positive charge is transferred to this oxygen. The acetate groups in the acetic anhydride are easily substituted to form sulfoester groups and the cellulose triacetate is produced as shown in Fig. 3. The reaction was carried out at a temperature less than 50 °C and the cellulose triacetate is produced. Due to the hydrolysis of the cellulose in acidic media, a reduction in the DS of the cellulose has occurred to obtain cellulose diacetate. The rate of the reaction as well as the extent of cellulose degradation, was dependent on the amount of acid and the reaction temperature (55 °C)^29^.

### Degree of substitution of nanocellulose acetate and elucidation of the structure

**Figure 4:**
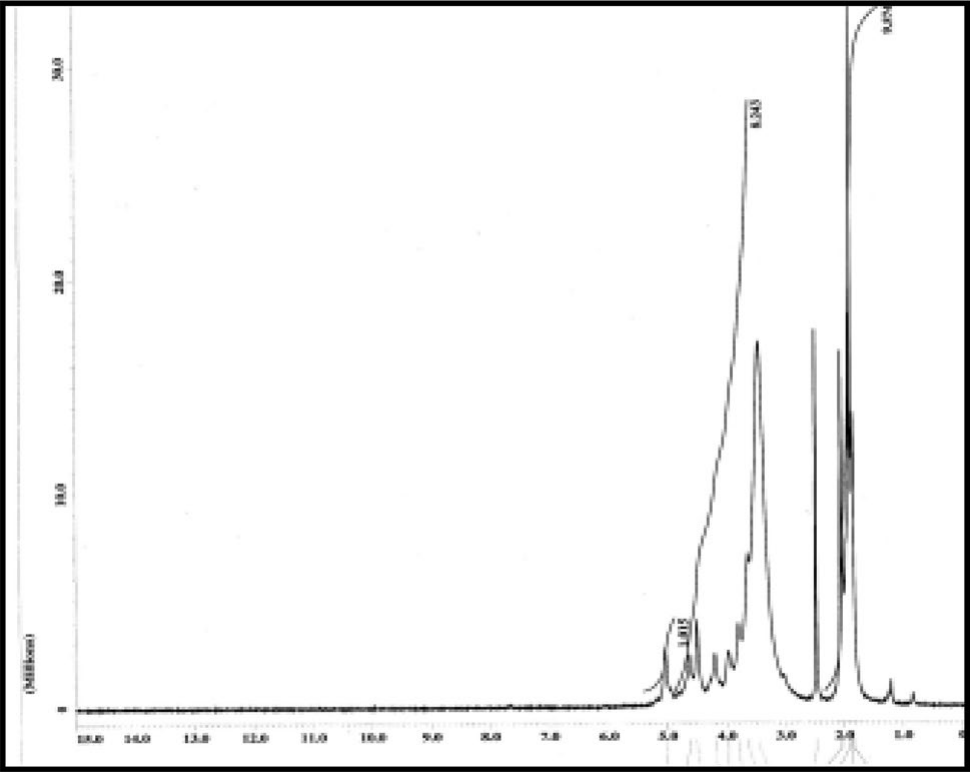
^1^H-NMR of Nanocellulose acetate prepared from rice.

The degree of substitution (DS) can be described as the extent to which the hydroxyl groups are substituted. The DS of CDA was determined by ^1^HNMR analysis of CA. The relative DS was estimated from the ratio between the peak heights or peak areas assigned to specific protons of certain groups appearing at different chemical shifts. Figure 4 shows the ^1^HNMR of the acetylated cellulose. It was observed that the ratio of the integrated two methyl protons of the acetyl group resonating between 1.92 and 2.11 ppm was compared to the integration of the total protons of cellulose absorbance between 3.5 and 5.1 ppm^30^.

The integrated value of the methyl protons in the acetyl groups was 8.3939, whereas the integrated value of the total ring protons was set to 7.705 This indicates that the DS from the Eq. (3) equals to:

$${\text{DS }} = \frac{7 \times 8.3939}{{3 \times 7.705}} = { 2}.{54}$$

Then the degree of substitution of The DS of the cellulose acetate produced by Nanorice was 2.54 proved that it was cellulose diacetate.

### The chemical structure

**Figure 5:**
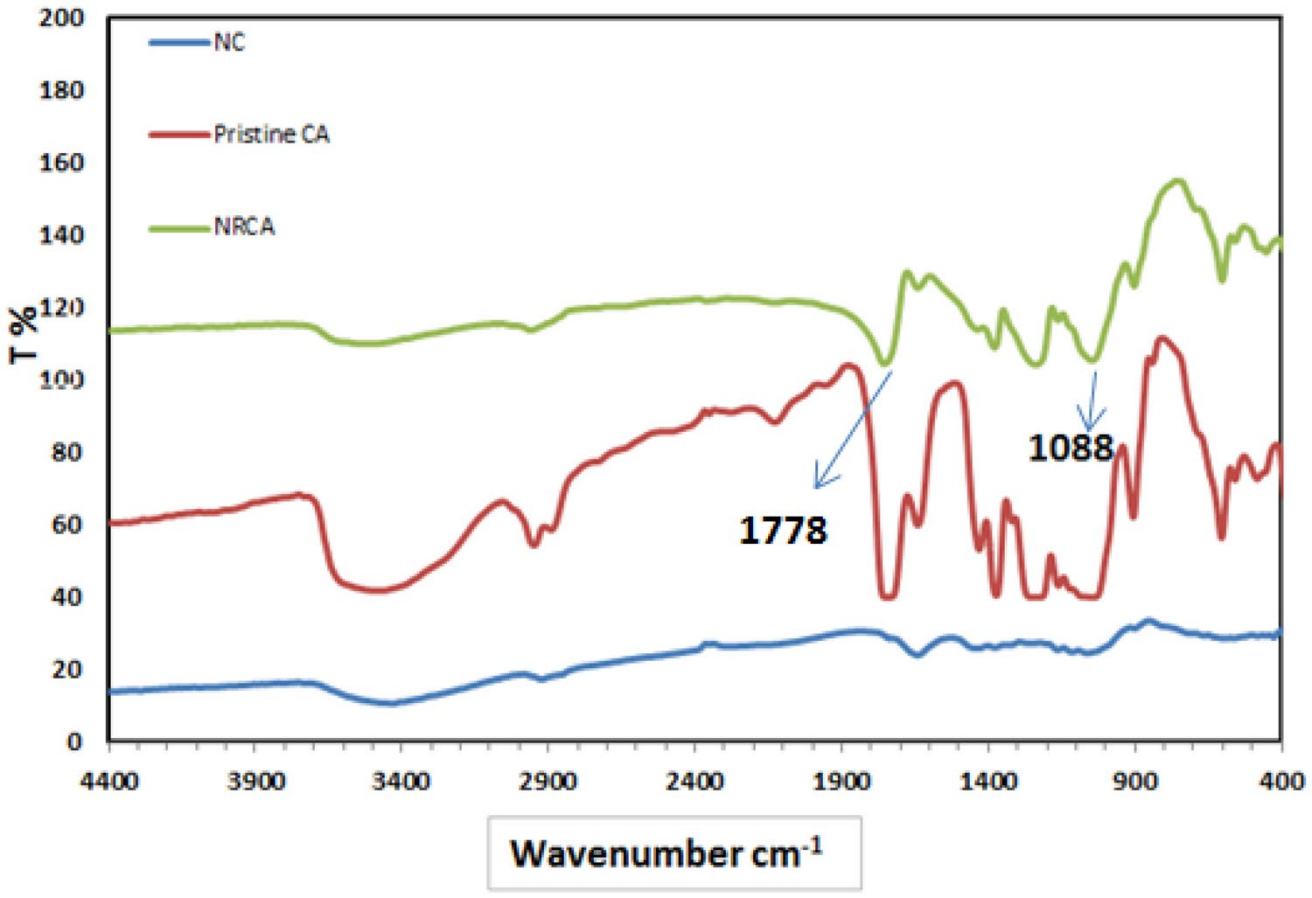
FTIR spectrum of Nanocellulose acetate compared with pristine cellulose acetate and Nanocellulose powder.

The chemical structure of the prepared NCA was deduced from the group frequencies within the infrared spectra. The FTIR spectra of the Nanocellulose and Nanocellulose diacetate extracted from rice straw were compared to the pristine cellulose acetate as shown in Fig. 5. The FTIR of NC shows that a broad absorption band resonating at 3300–3680 cm^−1^ is attributed to the stretching vibration of the OH groups. Furthermore, a peak at 2990 cm^-1^ corresponded to the C–H stretch of alkane. The absorption bands at 1595 and 1510 cm^-1^ are assigned to C=C and C–O stretching, respectively^24,27^.

A broad absorption band resonating at 3450–3590 cm^−1^ is attributed to the hydroxyl groups (OH) in the pristine cellulose acetate. The band at 2992 cm^−1^ is attributed to the CH_3_ stretching vibration. In addition, a peak at 1755 cm^−1^ corresponded to the carbonyl group (C=O), while the band at 1244 cm^−1^ corresponded to the stretching modes of the symmetric (C–O–C) bond of the cellulosic esters^31^.

Nanocellulose diacetate preparation was confirmed by the appearance of the band at 1778 cm^−1^ of the C=O of the acetyl carboxylic group and that at 1090 cm^−1^ of the asymmetry C–O–C stretching bands of the dimers. There is a broad absorption band resonating at 3528 cm^−1^ corresponding to the stretching vibration of the OH groups. The peak intensities of the acetate group at 1753 cm^−1^ were correlated to the degree of substitution of nanocellulose acetate, as the absorption intensity of this peak has increased when the degree of substitution was increased^32^.

### Crystallinty within the extracted CA (X-ray Diffraction)

**Figure 6:**
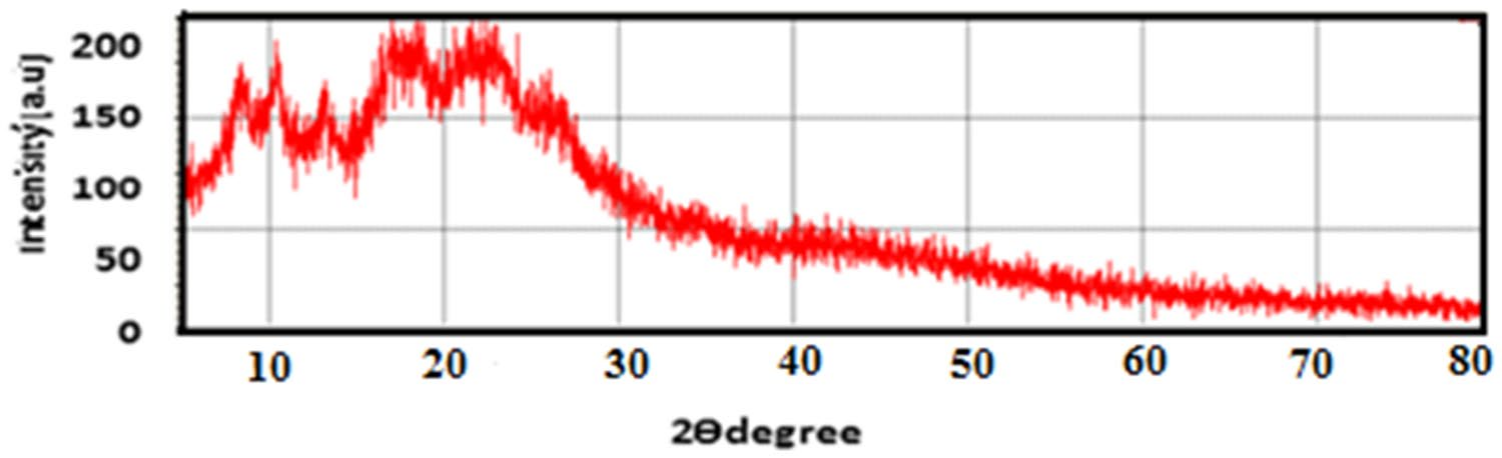
XRD patterns Nanocellulose acetate prepared from rice straw.

The cellulose molecules had extensive hydrogen bonding between the cellulose chains, producing a strong crystalline structure. X-ray diffraction patterns have been a powerful tool to investigate the degree of crystalinity within the polymer chains. The XRD patterns of NCA was recorded in the range of 2θ = 0–80° as shown in Fig. 6. The XRD pattern of the NCA had two broad peaks at 10° and 20° indicating that the NCA is mainly transparent and had an amorphous structure with minor crystalline regions. CA had a certain ratio of amorphous and crystalline phases organized^33,34^.

The acetylated product of CA is a less crystalline material, which indicates the generation of a molecular disorder that was caused by the substitution of the acetyl groups that have a greater volume along the axes and is thus associated with an increase in the inter distance ^35,36^.

### Morphological properties of prepared CA-RO membranes

**Figure 7:**
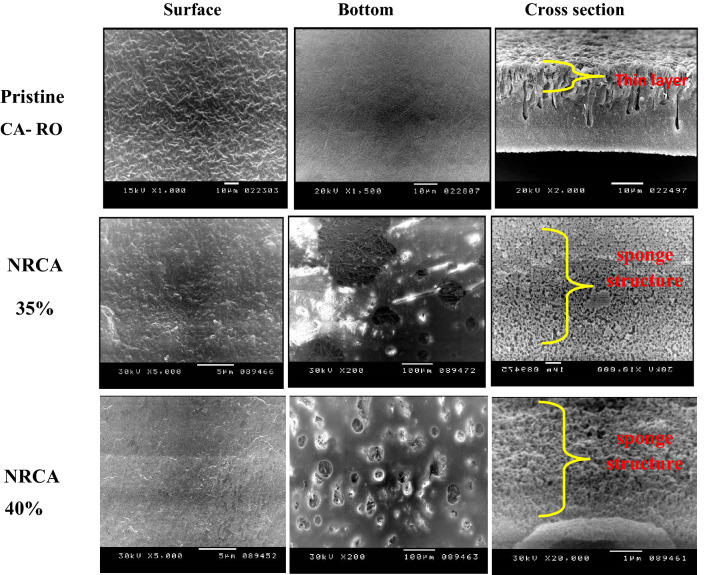
SEM images of surface, bottom and cross section of NRCA membranes compared with pristine CA-RO membrane.

The Morphology of the surface of the CA-RO membranes was illustrated the thin skin layer of CA is formed instantly at the top of the cast film due to the loss of solvent. This thin layer of CA that's forming during the first evaporation step becomes the top skin layer governs the permeation properties of the asymmetric membrane, while the porous structure that's formed during the solvent-nonsolvent extraction step becomes the porous sublayer^29^. A number of factors have the tendency to create macro voids structures, others help in suppressing the macro voids improving the interconnectivity of the pores, and leading to higher porosities within the top layer and also the sub-layer. The Structure and properties of membranes prepared by the phase inversion method depend upon many factors such as solvent-non solvent type, the temperature of annealing, molecular weight, type of additives. Either enlargement or suppression of macro void can be controlled by these factors. In the finger-like structured membranes, there is a dense skin layer on the uppermost surface of the membrane and the tapering finger cavities form single pores from the top to almost the bottom surface of the membrane. This result is identical to those produced by Matsuura et al.^19^. The pristine CA-RO membrane displays a relatively high surface roughness. The smoother surface membranes were shown in the nanocellulose membrane prepared from Rice. Fouling often links to the intrinsic membrane properties as the fouling rates increase with the increase of the membrane surface roughness because foul ant particles are more likely to be entrained by rougher topologies than by smoother membrane surfaces^29^. Generally, large finger-like cavities in the membrane are formed when the cast solution precipitates rapidly; Meanwhile the pore structure is reduced. On the other hand, the sponge structure of the membrane is formed and becomes dominant when the precipitation process was slow as shown in Figs. 7 NCCA:CA35:65 membranes and NCCA: CA40:60 membranes. The ridge-and-valley morphology is observed from the surface of pristine CA-RO membrane (Fig. 7). The surface image for pristine CA-RO membrane, pinholes have appeared and the dense top layer which was formed is supported by a bottom layer containing pores. Figure 7 show the cross-sectional, surface, and bottom structures of membranes. It is seen from the figures that the prepared CA-RO membranes are having asymmetric structures consisting of a dense top layer and a porous sublayer. Due to the addition of nanocellulose leading to the formation of finger-like cavities in the sublayer of the prepared membranes^37^.

### Hydrophilic properties of nano CA-RO membranes

**Figure 8:**
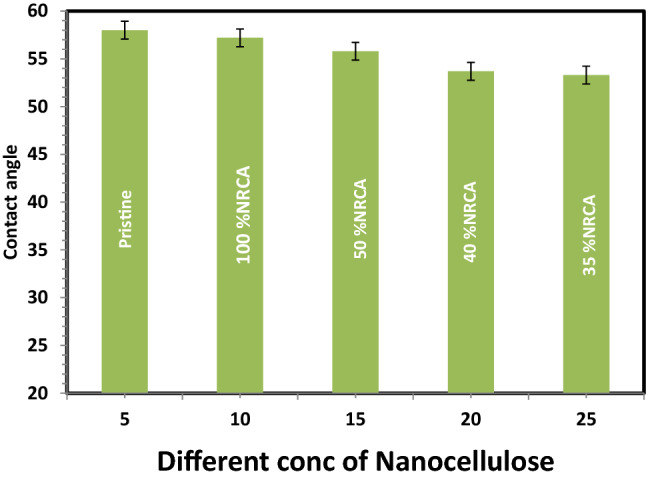
Contact angle of different concenteration nanocellulose membrane versus pristine CA membrane.

Membrane hydrophobicity was determined by measuring the contact angle between the membrane surface and a water droplet as shown in Fig. 8. Contact angle measurements were obtained through the captive bubble technique^37^. The hydrophilic property is an interesting property to be measured and it is correlated to the surface energy of the membrane. This makes the surface energy together with surface roughness one of the most interesting properties to consider when working with fouling membranes. This is especially true when proteins and other biologically active substances are present in the solution to be filtered. The surface hydrophilicity of the prepared CA-RO membranes can be evaluated through contact angle measurements. The combination of relatively smooth, hydrophilic, and negatively charged film layers typically produces better water permeability, salt rejection, and fouling resistance in water purification. Figure 8 show as the concentration of nanocellulose increase the contact angle slightly decrease so relative improvement in hydrophilicity is achieved by the addition of nanocellulose acetate and this data is similar to that achieved by Liu et al.^14^. The contact angles were 58° and 53.3° corresponding to the pristine CA and nano cellulose acetate membranes respectively.

### Salt rejection and water flux of the nano CA-RO membranes

**Figure 9:**
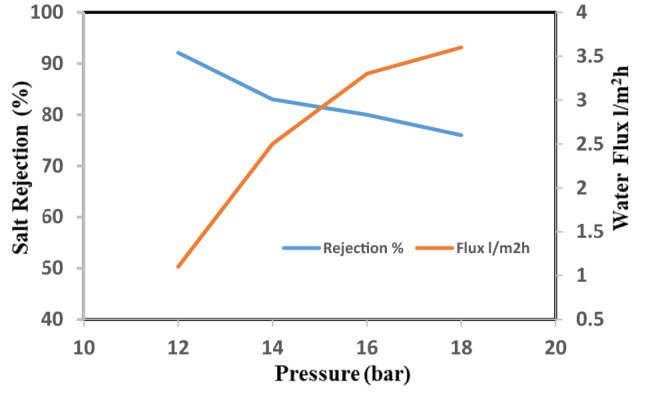
Salt rejection and water flux versus feed pressure of pristine CA-RO membrane.

This study discussed the effect of different concentrations of nanoparticles on membrane performance. Figure 9 show the salt rejection and water flux for the CA-RO membranes the salt rejection for pristine CA-RO membrane is 92.1% and water flux of 1.1 L/m^2^h at 12 bar, which decreased to 76.0% at 18 bar and the salinity increases at the membrane surface, the local osmotic pressure will be increased attributed to the effect of the concentration polarization as a boundary layer formed near the membrane surface at which the salt concentration exceeds in the bulk solution. Meanwhile, the increase in salinity at the membrane surface increases the salt transport through the membrane due to the overall pressure difference between the hydrostatic pressure and the osmotic pressure decreases, and thus the permeate flow is reduced.

**Figure 10:**
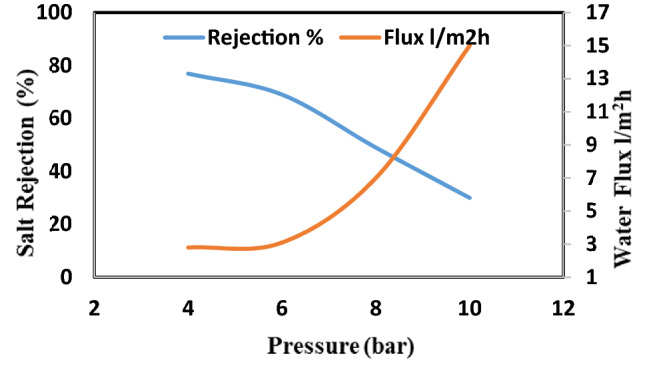
Salt rejection and water flux versus feed pressure of CA-RO membrane with 100% NRCA membrane.

**Figure 11:**
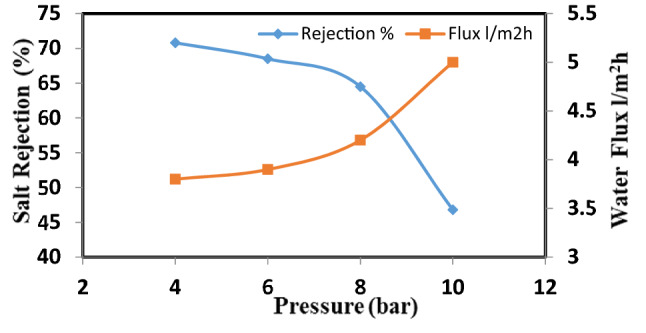
Salt rejection and water flux versus feed pressure of CA-RO membrane with 50% NRCA membrane.

**Figure 12:**
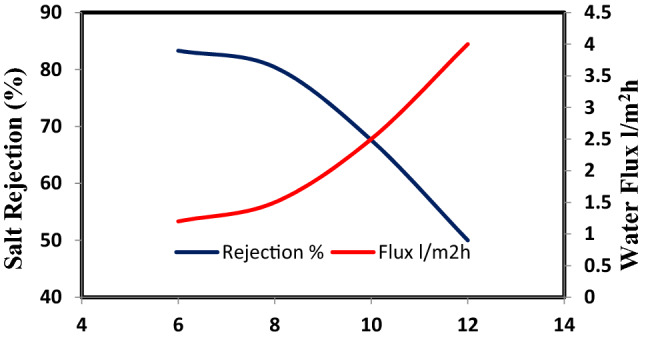
Salt rejection and water flux versus feed pressure of CA-RO membrane with 35% NRCA membrane.

**Figure 13:**
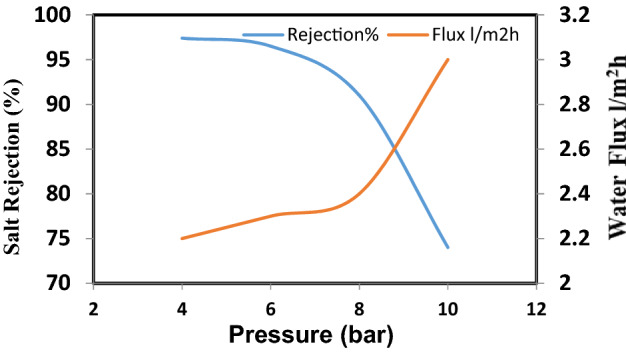
Salt rejection and water flux versus feed pressure of CA-RO membrane with 40% NRCA membrane.

Nanocellulose acetate extracted from Rice straw was added to cellulose acetate membrane solution to study the effect of Nanocellulose acetate powder on the performance of membranes throw salt rejection and water flux. Remarkable improvement in membrane flux properties is achieved by the addition of nanocellulose acetate and this data is identical to that achieved by Liu et al.^14^. Figures 10, 11, and 12 show the distribution of nanoparticles at small concentrations is almost uniform, without any big aggregation effect. However, increasing the amount of nanoparticles in the membrane matrix leads to aggregate formation. Thus a reduced affected area of newly formed aggregates may cause decrease in salt rejection. While the a decrease of Nanocellulose to 40% has a positive effect on salt rejection due to the formation of finger-like structure which has a positive effect on salt rejection (Fig. 13).

Generally adding nanocellulose to cellulose acetate membrane improve hydrophilicity and water flux^14^, but adding 40% nanocellulose acetate in Fig. 13 it’s the optimum concentration has the highest water flux of 2.2 and increase the salt rejection to 97.4% according to the solution and diffusion model, the flux was proportional to the net differential pressure across the membrane and this results as presented in Table 2.

**Table 2:** Effect of concentration nano cellulose.

Conc. of nanocellulose acetate membranes	Salt rejection %	Flux l/m^2^ h
100%CA	93	1.1
100% RNCA	76.9	2.8
50: 50% RNCA:CA	70.8	3.8
40:60% RNCA: CA	97.4	2.2
35:65% RNCA:CA	83.3	1.1

### Antibiofouling of nanocellulose acetate membranes

**Figure 14:**
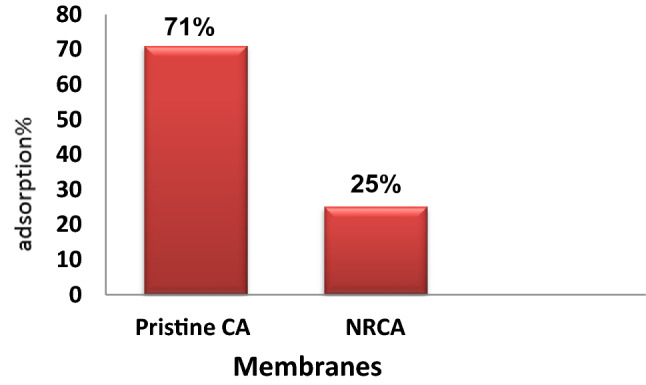
The albumin adsorption on the Nanocellulose and pristine cellulose acetate membrane.

The ability to resist protein adsorption could be a prerequisite for a surface to resist microbial adhesion. Furthermore, the adsorption of proteins to a membrane usually demonstrates a good correlation with the anti-biofouling property of a hydrophobic membrane. Figure 14 shows the protein adsorption of prepared membranes. As clearly seen, the membranes prepared with an addition of nanocellulose showed significantly higher resistance toward adsorptive fouling than the pristine membrane as noticed by their much lower RP%. It was found that the relative protein adsorption of pristine membrane decreased in the casting solution from 71 to 25% with the addition of nanocellulose. As seen, the membranes containing nanocellulose seed extract have lower RP%; this result can be explained by the enhancement membrane surface contributed to the formation of hydration layers via ionic solvation of the charged groups and hydrogen bonds between the amide groups and therefore the water molecules^38,39^. The hydration layers led to a robust force to protein at a particular distance and made the protein in contact with the membrane surface in a very reverse manner^40^. Consequently, the protein adsorption is proscribed to development membrane surfaces.

## Conclusions

Cellulose was successfully extracted and Nanocellulose acetate was prepared from rice straw as an agricultural waste using the acid hydrolysis technique. The SEM images of the prepared Nanocellulose revealed that the particle size was about 22 nm. It was found that the prepared Nanocellulose acetate had a DS 2.54 for CDA from HNMR. This shows that acetylation of rice straw can be a simple procedure to develop an inexpensive and biodegradable Nanocellulose acetate which can effectively replace raw materials from fossil fuels as a starting material in polymers industries. Development membrane with nanocellulose could resist effectively protein adsorption and microbial growth; therefore, they should be considered as additives in practical applications.

## Data Availability

The datasets generated during and analyzed during the current study are not publicly available due to (Because it falls within the intellectual property rights of the financiers) but are available from the corresponding author on reasonable request.
